# Supplementary material for: nNOS-expressing neurons in the vmPFC transform pPVT-derived chronic pain signals into anxiety behaviors
Source: Nat Commun. 2020 May 19;11:2501. doi: 10.1038/s41467-020-16198-5 (PMC7237711; doi:10.1038/s41467-020-16198-5)
Supplement: Supplementary file 1 — Supplementary Information [file 41467_2020_16198_MOESM1_ESM.pdf]

SUPPLEMENTARY INFORMATION

nNOS-expressing neurons in vmPFC transform pPVT-derived chronic pain signals to anxiety behaviors

Liang *et al*.

Supplementary Table 1 Key resources table

REAGENT or RESOURCE	SOURCE	IDENTIFIER
Antibodies		
Primary antibodies		
Rabbit anti-nNOS, polyclonal (IF, 1:400; WB, 1:2000)	Thermo Scientific	Cat# 61-7000; RRID: AB_2313734
Guinea pig anti-c-Fos, polyclonal (IF, 1:200)	Synaptic System	Cat# 226-004; RRID: AB_2619946
Chicken anti-GFP, polyclonal (IF, 1:1000)	Millipore	Cat# AB16901 ; RRID: AB_90890
Rabbit anti-GFP, polyclonal (IF, 1:1000)	Abcam	Cat# ab290 ; RRID: AB_303395
Mouse anti-mCherry, monoclonal (IF, 1:100)	Abcam	Cat# ab125096; Clone 1C51; RRID: AB_11133266
Rabbit anti-CaMKII alpha, polyclonal (IF, 1:50)	Abcam	Cat# ab103840; RRID: AB_10900968
Mouse anti-vGAT, monoclonal (IF, 1:100)	Synaptic System	Cat# 131011; Clone 117G4; RRID: AB_887872
Rabbit anti-GluA1, monoclonal (WB, 1:4000)	Abcam	Cat# ab109450; Clone EPR5479 RRID: AB_10860361
Rabbit anti-GluA2, monoclonal (WB, 1:4000)	Abcam	Cat# ab133477; Clone EPR5032 RRID: AB_2620181
Rabbit anti-stargazin, polyclonal (WB, 1:1000)	Millipore	Cat# 07-577; RRID: AB_310726
Rabbit anti-NeSF, polyclonal (WB, 1:2000)	Abcam	Cat# ab87155; RRID: AB_10674097
Mouse anti-GAPDH, monoclonal (WB, 1:8000)	KangChen Bio-tech	Cat# KC-5G4; Clone 6C5; RRID: AB_2493106
Secondary antibodies		
Cy3 goat anti-rabbit (IF, 1:400)	Jackson ImmunoResearch Laboratories	Cat# 111-165-003; RRID: AB_2338000
Alexa 488 goat anti-guinea pig (IF, 1:200)	Abcam	Cat# ab150185; RRID: AB_2736871
Alex 488 goat anti-chicken (IF, 1:200)	Jackson ImmunoResearch Laboratories	Cat# 103-545-155; RRID: AB_2337390
Cy3 goat anti-mouse (IF, 1:200)	Jackson ImmunoResearch Laboratories	Cat# 115-165-003; RRID: AB_2338680
Alexa 488 Goat anti-rabbit (IF, 1:100)	Jackson ImmunoResearch Laboratories	Cat# 111-545-003; RRID: AB_2338046
Goat Anti-mouse HRP (WB, 1:8000)	Multi Science	Cat# 70-GAM0072; RRID:AB_2827834

(continued on next page)

Continued

REAGENT or RESOURCE	SOURCE	IDENTIFIER
Goat Anti-rabbit HRP polyclone (WB, 1:4000~1:6000)	Multi Science	Cat# 70-GAR0072;
**Recombinant Virus Strains**		
AAV-hSyn-HA-hM4Di-IRES-mCitrine	Genechem	Cat# AAV00093
AAV-CaMKIIα-hM4Di-mCherry	BrainVTA	Cat# PT-0017
AAV-CaMKIIα-hM3Dq-mCherry	BrainVTA	Cat# PT-0049
AAV-CaMKIIα-mCherry	BrainVTA	Cat# PT-0108
AAV-CaMKIIα-eGFP	Genechem	Cat# AAV00083
AAV-hsyn-DIO-hM3Dq-eGFP	Genechem	Cat# AAV00071
AAV-hsyn-DIO-hM4Di-eGFP	Genechem	Cat# AAV00099
AAV-hsyn-DIO-mCherry	Genechem	Cat# AAV00061
AAV-hSyn-DIO-eGFP	OBiO	Cat# HYMBH4883
AAV-hsyn-DO-hM3Dq-eGFP	BrainVTA	Cat# PT-2155
AAV-EF1a-DIO-ChR2-eYFP	BrainVTA	Cat# PT-0001
AAV-CaMKIIα-ChR2-eYFP	Genechem	Cat# AAV00012
AAV-CMV-flex-shVGAT-eGFP	Genechem	N/A
AAV-CMV-flex-shVGLUT-eGFP	Genechem	N/A
AAV-CMV-flex-shControl-eGFP	Genechem	N/A
**Chemical and Biochemical Reagents**		
Complete Freund's adjuvant (CFA)	Sigma-Aldrich	Cat# F5881
Clozapine-N-Oxide (CNO)	Tocris Bioscience	Cat# 4936; CAS number: 34233-69-7
D-(-)-2-Amino-5-phosphonopentanoic acid (AP-5)	Tocris Bioscience	Cat# 0106; CAS number: 79055-68-8
2-(4-Carboxyphenyl)-4,4,5,5-tetramethy limidazoline-1-oxyl-3-oxide (C-PTIO)	Sigma-Aldrich	Cat# C221; CAS number: 148819-94-7
N^5-(1-imino-3-butenyl)-L-ornithine (L-VNIO)	Alexis Biochemicals	Cat# 270-216; CAS number: 728944-69-2
6-cyano-7-nitroquinoxaline-2,3-dioneis (CNQX)	Sigma-Aldrich	Cat# C239; CAS number: 115066-14-3
(-)-Bicuculline methobromide (BMI)	Tocris Bioscience	Cat# 0109; CAS number: 73604-30-5
3,3-bis(aminoethyl)-1-hydroxy-2-oxo-1-triazene (DETA/NONOate)	Sigma-Aldrich	Cat# A5581; CAS number: 146724-94-9
Tetrodotoxin (TTX)	Sigma	Cat# T8024 ; CAS number: 4368-28-9
4-aminopyridine (4-AP)	Tocris Bioscience	Cat# 0940; CAS number: 504-24-5
Diphenyleneiodonium chloride	Millipore	Cat# 300260; CAS number: 4673-26-1

(continued on next page)

Continued

REAGENT or RESOURCE	SOURCE	IDENTIFIER
1400W	Sigma-Aldrich	Cat# W4262 ; CAS number: 214358-33-5
Hoechst 33258 (HOE)	Sigma-Aldrich	Cat# B1155; CAS number: 23491-45-4
HPDP-biotin	Thermo Scientific	Cat# 21341
Avidin-affinity resin beads	Sigma-Aldrich	Cat# 85881
Critical Commercial Assays		
Nitric Oxide Assay Kit	Beyotime Biotech	Cat# S0023
RNAscope assay kit	Advanced Cell Diagnostics	Cat# 323100
RNAscope® Probe-Mm-Nos1	Advanced Cell Diagnostics	Cat# 437651
RNAscope® Probe-EGFP-C2	Advanced Cell Diagnostics	Cat# 400281-C2
Mouse Strains		
C57BL/6JNju	Model Animal Research Center of Nanjing University	Cat# N000013
B6.129-*Nos1*$^{tm1(cre)Mgmj}$/J (nNOS-Cre)	The Jackson Laboratory	Cat# 017526; RRID: IMSR_JAX:017526
Software and Algorithms		
GraphPad Prism 6 software	GraphPad Software, Inc.	https://www.graphpad.com/ ; RRID: SCR_002798
pClamp 10	Molecular Devices	http://mdc.custhelp.com/ ; RRID: SCR_011323
Mini Analysis Program 6.0	Synaptosoft, Inc.	http://www.synaptosoft.com/MiniAnalysis/; RRID: SCR_002184
TopScan LITE software	Clever Sys, Inc.	http://www.cleversysinc.com RRID: SCR_014494
ZEN2009 Light Edition	Carl Zeiss	https://www.zeiss.com/ ; RRID: SCR_013672
Adobe Photoshop CS6	Adobe Systems	https://www.adobe.com/ ; RRID: SCR_014199
Image Lab (Beta 7)	Bio-Rad Laboratories	http://www.bio-rad.com/en-us/sku/1709690-image-lab-software/ ; RRID: SCR_014210
ImageTool 3.0	UTHSCSA	http://uthscsa-imagetool.software.informer.com/ ; RRID:SCR_016208

Abbreviates: IF, immunofluorescence; WB, western blot.

Supplementary Figures

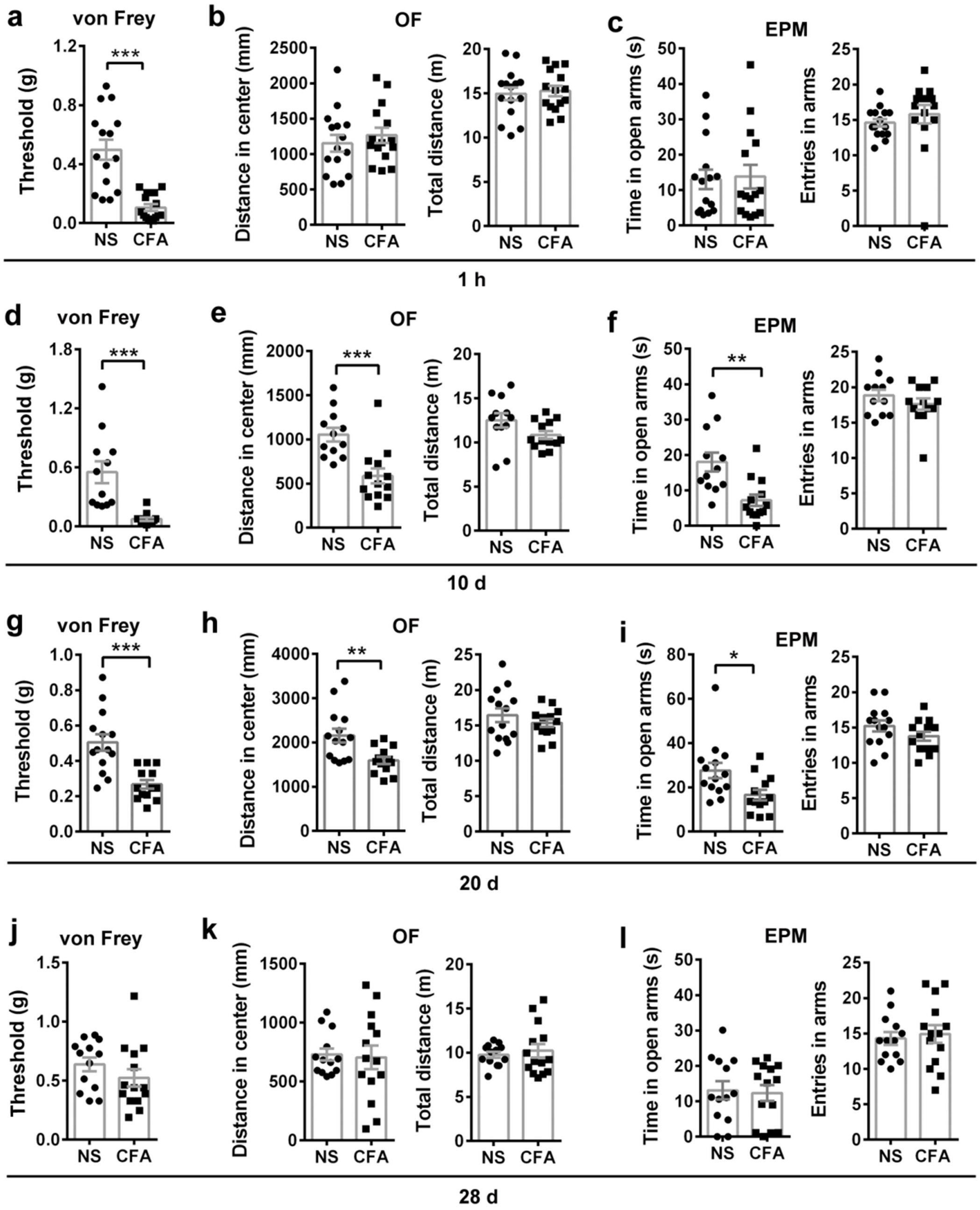

Supplementary Fig. 1 CFA-induced chronic pain and anxiety-like behaviors. **a-c** Withdrawal threshold of the hindpaw in response to von Frey hair stimulation (**a**), distance in center (**b**, left) and total distance (**b**, right) in OF, time in open arms (**c**, left) and total entries in arms (**c**, right) of EPM, 1 h after CFA (10 μl) or NS (10 μl) injection. n = 15. **d-f** Withdrawal threshold of the hindpaw in von Frey test (**d**), distance in center (**e**, left) and total distance (**e**, right) in OF, time in open arms (**f**, left) and total entries in arms (**f**, right) of EPM, at day 10 after CFA or NS injection. n = 12 for NS and n = 13 for CFA. **g-i** Withdrawal threshold of the hindpaw in von Frey test (**g**), distance in center (**h**, left) and total distance (**h**, right) in OF, time in open arms (**i**, left) and total entries in arms (**i**, right) of EPM, at day 20 after CFA or NS injection. n = 14 for NS and n = 13 for CFA. **j-l** Withdrawal threshold of the hindpaw in von Frey test (**j**),

distance in center (k, left) and total distance (**k**, right) in OF, time in open arms (**l**, left) and total entries in arms (**l**, right) of EPM, at day 28 after CFA or NS injection. $n = 13$ for NS and $n = 14$ for CFA. Data are mean ± SEM; $*p < 0.05$, $**p < 0.01$, $***p < 0.001$ (unpaired two-tailed Student's t-test). Source data are provided as a Source Data file. Exact p values and additional statistical information can be found in Source data.

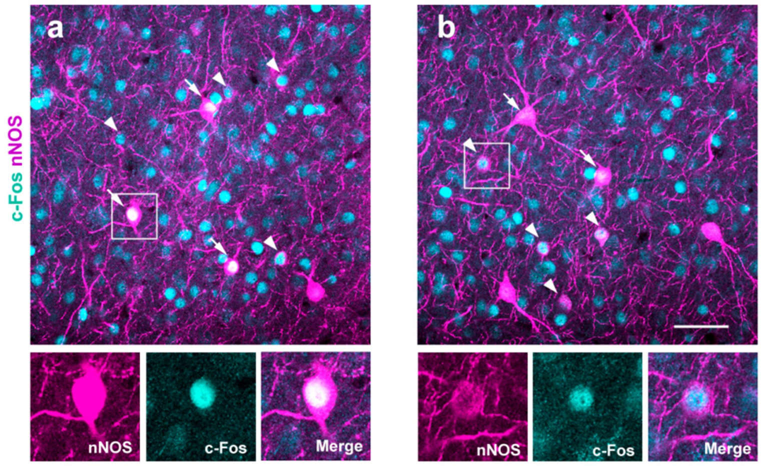

Supplementary Fig. 2 Immunofluorescent images showing nNOS-expressing neurons in vmPFC co-labeled with c-Fos 4 h after CFA injection. Arrows marked type I and arrowheads marked type II nNOS-expressing neurons. Lower panels are magnified images of selected areas indicating a type I (strongly labeled) (**a**) and a type II (weakly labeled) **(b)** nNOS-expressing neurons. Similar results were observed in 6 mice. Scale bar, 50 μm.

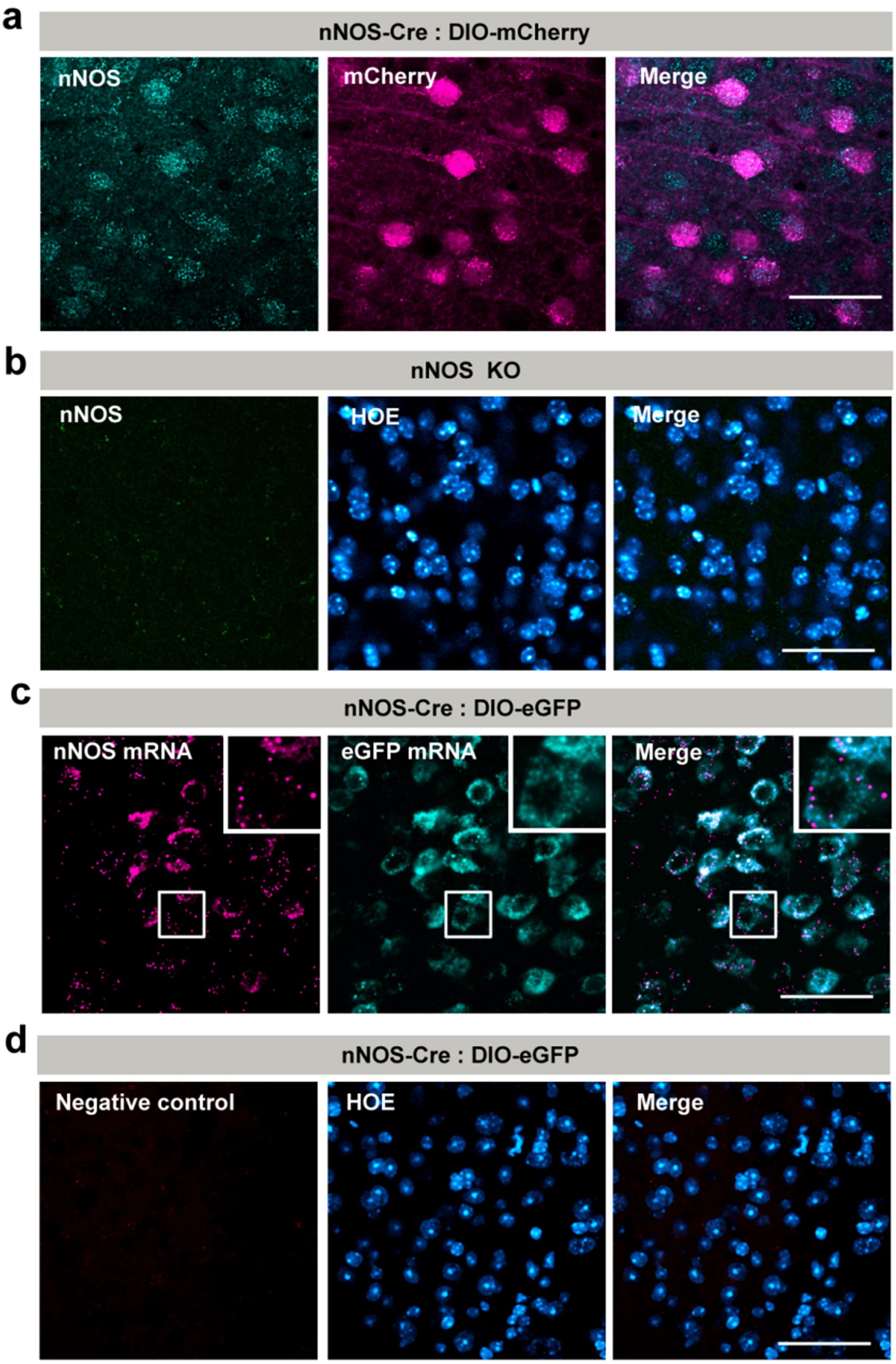

Supplementary Fig. 3 Cre-dependent expression in vmPFC nNOS neurons. **a** Immunofluorescent images showing Cre-dependent expression of mCherry in type II

nNOS-expressing neurons (arrowheads indicated) 3 weeks after AAV-hSyn-DIO-mCherry (0.4 μl) was microinjected in vmPFC of nNOS-Cre mice. About 96% mCherry$^+$ neurons were nNOS positive. Similar results were observed with 4 mice in triplicate experiments. **b** Immunofluorescent images showing no positive labeling with nNOS antibody in vmPFC of nNOS KO mice. Similar results were observed with 3 mice in triplicate experiments. **c** Images of fluorescence in situ hybridization (FISH) showing colocalization of nNOS mRNA and Cre-dependent eGFP mRNA in vmPFC. About 92% eGFP mRNA$^+$ neurons were nNOS mRNA positive. Similar results were observed with 5 mice in triplicate experiments. **d** FISH image labeled with negative control probe. Similar results were observed with 3 mice in triplicate experiments. Scale bar, 50 μm.

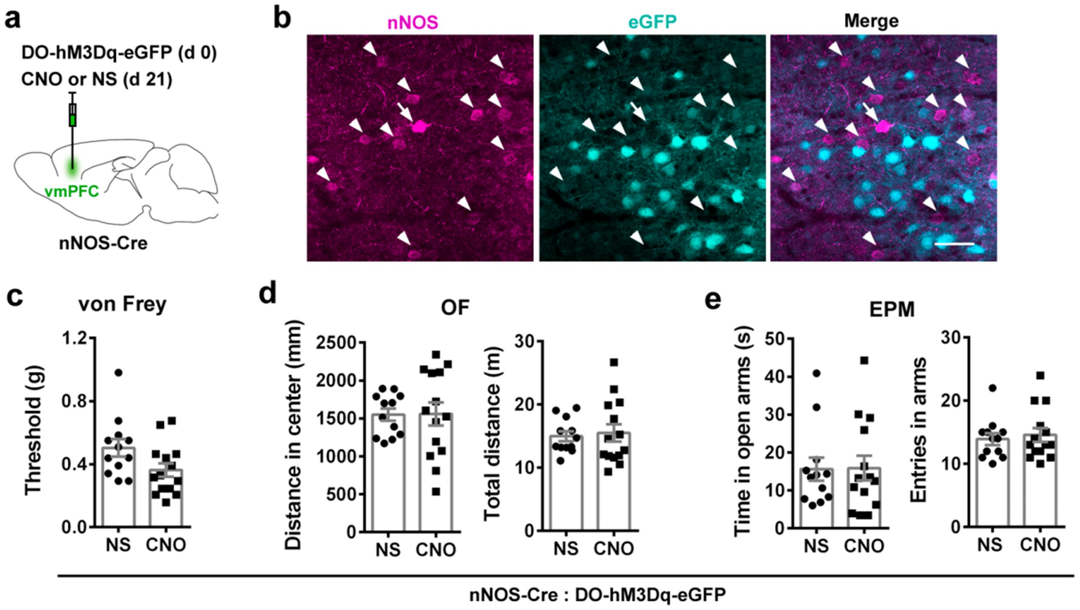

Supplementary Fig. 4 Chemogenetic excitation of nNOS negative neurons in vmPFC had no effects on the anxiety-like behaviors induced by chronic pain. **a** Scheme indicating the vmPFC area where AAV-hSyn-DO-hM3Dq-eGFP (0.4 μl) and CNO (0.5 mM, 1 μl) or NS (1 μl) was microinjected into nNOS-Cre mice. **b** Immunofluorescent images indicating expression of hM3Dq-eGFP in vmPFC nNOS negative neurons. Arrows marked type I and arrowheads marked type II nNOS-expressing neurons. Similar results were observed in 3 mice. **c-e** Withdrawal threshold of the hindpaw in von Frey test (**c**), distance in center (**d**, left) and total distance (**d**, right) in OF, time in open arms (**e**, left) and total entries in arms (**e**, right) of EPM. nNOS-Cre mice were microinjected AAV-hSyn-DO-hM3Dq-eGFP 21 days before behavioral tests. n = 12 for NS, and n = 14 for CNO. Data are mean ± SEM; Scale bar, 50 μm. t_{24} = 2.040, p = 0.053 for **c**, t_{24} = 0.310, p = 0.760 for **d** (left), t_{24} = 0.057, p = 0.955 for **d** (right), t_{24} = 0.058, p = 0.954 for **e** (left), and t_{24} = 0.449, p = 0.658 for **e** (right) (unpaired two-tailed Student's t-test). Source data are provided as a Source Data file. Exact p values and additional statistical information can be found in Source data.

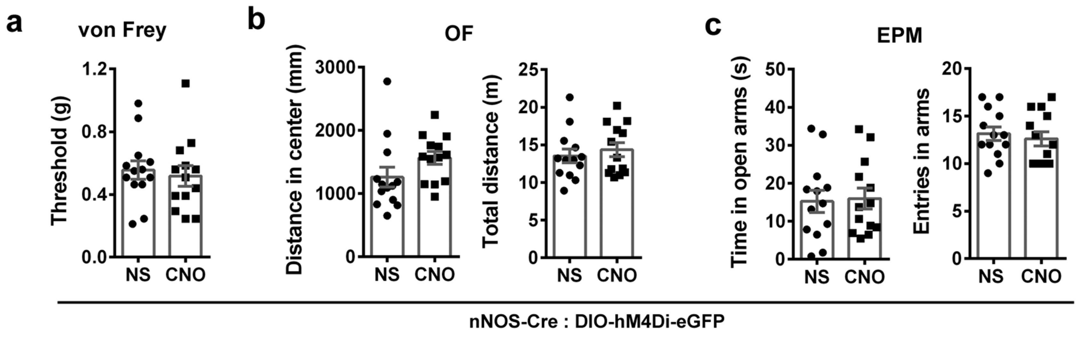

Supplementary Fig. 5 Chemogenetic inhibition of nNOS-expressing neurons in vmPFC did not produce significant anxiolytic effect in normal animals. **a-c** Withdrawal threshold of the hindpaw in von Frey test (**a**), distance in center (**b**, left) and total distance (**b**, right) in OF, time in open arms (**c**, left) and total entries in arms (**c**, right) of EPM. nNOS-Cre mice were microinjected AAV-hSyn-DIO-hM4Di-eGFP (0.4 μl) within vmPFC 21 days before behavioral tests. CNO (0.5 mM, 1 μl) or NS (1 μl) were microinjected into vmPFC 30 min before behavioral tests. $n = 13$. Data are mean ± SEM. $t_{24} = 0.423$, $p = 0.676$ for a, $t_{24} = 1.615$, $p = 0.119$ for **b** (left), $t_{24} = 0.631$, $p = 0.534$ for **b** (right), $t_{24} = 0.186$, $p = 0.854$ for **c** (left), and $t_{24} = 0.524$, $p = 0.605$ for **c** (right) (unpaired two-tailed Student's *t*-test). Source data are provided as a Source Data file. Exact *p* values and additional statistical information can be found in Source data.

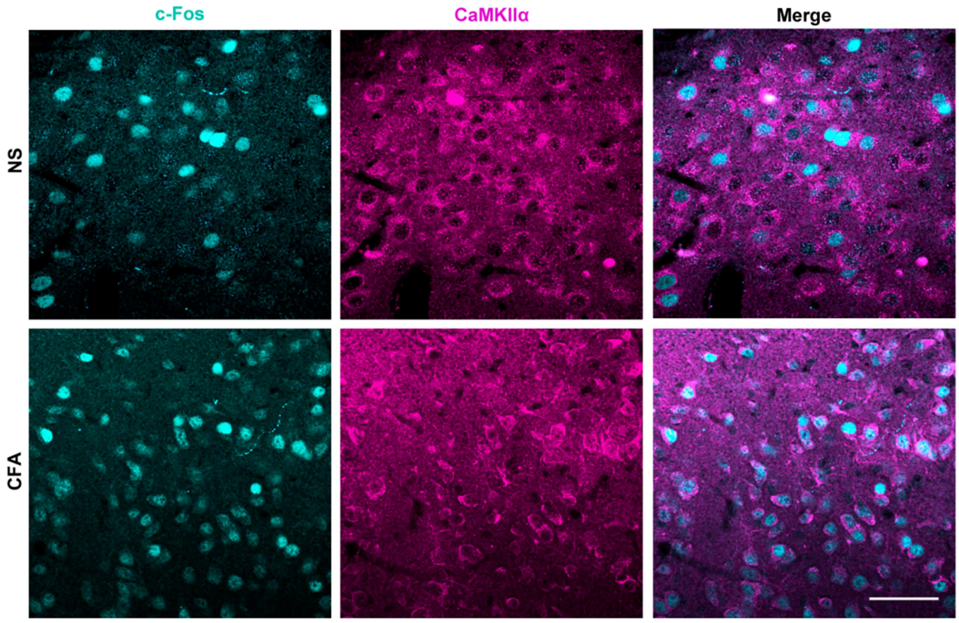

Supplementary Fig. 6 The activated neurons in the pPVT after CFA injection was glutamatergic. The immunofluorescent images illustrated c-Fos and CaMKIIα double labeled neurons in pPVT. Similar results were observed in 5 mice. Scale bar, 50 μm.

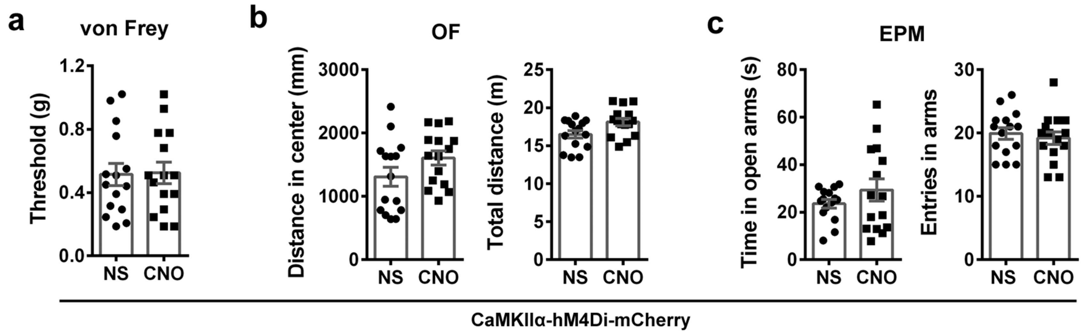

Supplementary Fig. 7 Chemogenetic inhibition of pPVT-vmPFC projections did not cause marked anxiolytic effect in normal mice. **a-c** Withdrawal threshold of hindpaw in von Frey test (**a**), distance in center (**b**, left) and total distance (**b**, right) of OF, and time in open arms (**c**, left) and total entries in arms (**c**, right) of EPM. Mice were microinjected AAV-CaMKIIα-hM4Di-mCherry (0.2 μl) within PVT 21 days before behavioral tests. CNO (0.5 mM, 1 μl) or NS (1 μl) were microinjected into vmPFC 30 min before behavioral tests. $n = 15$. Data are mean ± SEM. Source data are provided as a Source Data file. Exact p values and additional statistical information can be found in Source data.

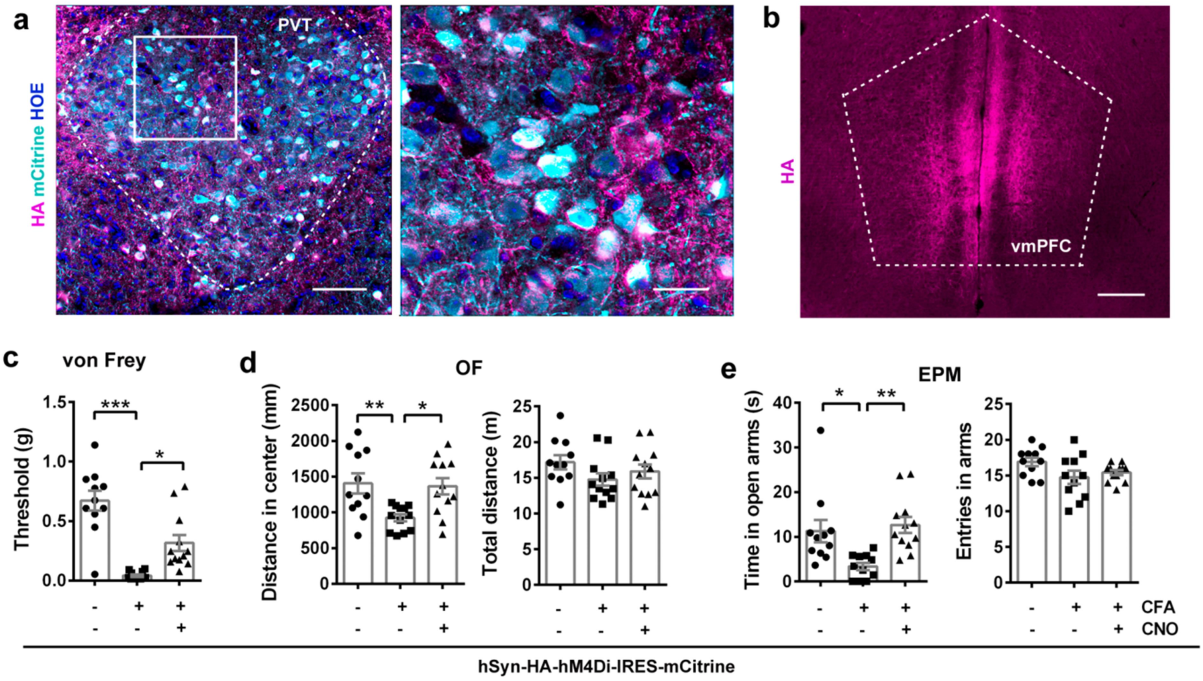

Supplementary Fig. 8 Inhibition of the pPVT-vmPFC projections by AAV-hSyn-HA-hM4Di-IRES-mCitrine attenuated CFA-induced chronic pain and anxiety-like behaviors. **a** Immunofluorescent images showing PVT neurons infected with AAV-hSyn-HA-hM4Di-IRES-mCitrine. Right is the magnified image of selected area in left. Similar results were observed in 3 mice. **b** Immunofluorescent image showing HA positive axon terminals in vmPFC from PVT. Similar results were observed in 3 mice. **c-e** Withdrawal threshold of hindpaw in von Frey test (**c**), distance in center (**d**, left) and total distance (**d**, right) in OF, time in open arms (**e**, left) and total entries in arms (**e**, right) of EPM. Experiment design was the same as that shown in Figure 3**k**, except that AAV-hSyn-HA-hM4Di-IRES-mCitrine was used instead of AAV-CaMKIIα-hM4Di-mCherry.

n = 11, 11 and 12, respectively. Data are mean ± SEM; *$p < 0.05$, **$p < 0.01$, and ***$p < 0.001$ (one-way ANOVA followed by Tukey's *post hoc* test). Scale bars, 100 μm in **a** (left), 25 μm in **a** (right) and 400 μm in **b**. Source data are provided as a Source Data file. Exact *p* values and additional statistical information can be found in Source data.

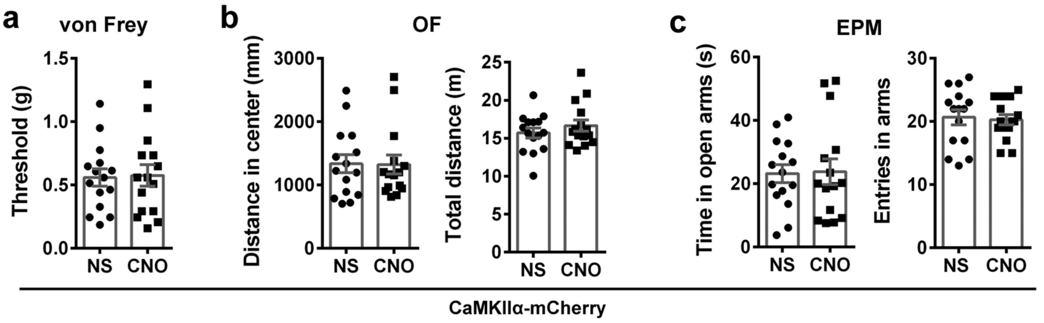

Supplementary Fig. 9 CNO did not change anxiety-like behaviors, without DREADDs. **a-c** Withdrawal threshold of hindpaw in von Frey test (**a**), distance in center (**b**, left) and total distance (**b**, right) of OF, and time in open arms (**c**, left) and total entries in arms (**c**, right) of EPM. Mice were microinjected AAV-CaMKIIα-mCherry (0.2 μl) within pPVT 21 days before behavioral tests. CNO (0.5 mM, 1 μl) or NS (1 μl) were microinjected into vmPFC 30 min before behavioral tests. *n* = 15. Data are mean ± SEM. Source data are provided as a Source Data file. Exact *p* values and additional statistical information can be found in Source data.

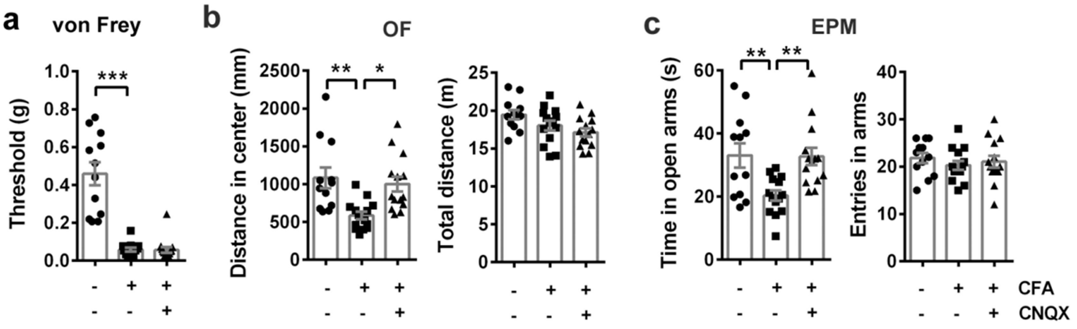

Supplementary Fig. 10 Blocking vmPFC AMPARs rescued CFA-induced anxiety-like behaviors. Bar graphs showing withdrawal threshold of hindpaw (**a**), distance in center (**b**) and total distance (**c**) of OF, and time in open arms (**d**) and total entries in arms (**e**) of EPM 30 min after CNQX (200 μM, 1 μl) or NS (1 μl) microinjection into vmPFC at day 3 after CFA or NS injection. *n* = 12, 14 and 14, respectively. Data are mean ± SEM; *$p < 0.05$ and **$p < 0.01$ (one-way ANOVA followed by Tukey's *post hoc* test). Source data are provided as a Source Data file. Exact *p* values and additional statistical information can be found in Source data.

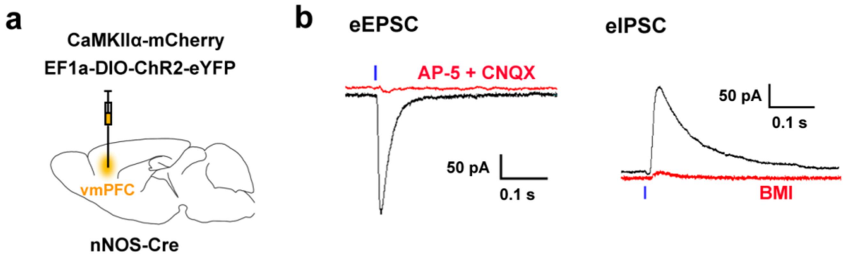

Supplmentary Fig. 11 vmPFC layer 2/3 pyramidal neurons were regulated by local nNOS-expressing neurons through both GABAergic and glutamatergic synapses. **a** Scheme indicating the vmPFC area where AAV-CaMKIIα-mCherry (0.2 μl) and AAV-EF1a-DIO-ChR2-eYFP (0.2 μl) were microinjected into nNOS-Cre mice. **b** Traces of light-evoked EPSC (left) and IPSC (right) recorded on layer 2/3 pyramidal neurons in vmPFC 3 weeks after the mixed AAVs were microinjected. This experiment was replicated in triplicate and similar results were obtained. $eYFP^+$ nNOS-expressing neurons were excited by blue light (465 nm, 43.7 mW, 5 ms duration) and $mCherry^+/eYFP^-$ pyramidal neurosns in layer 2/3 of vmPFC were recorded in the presence of TTX (0.5 μM) and 4-AP (100 μM).

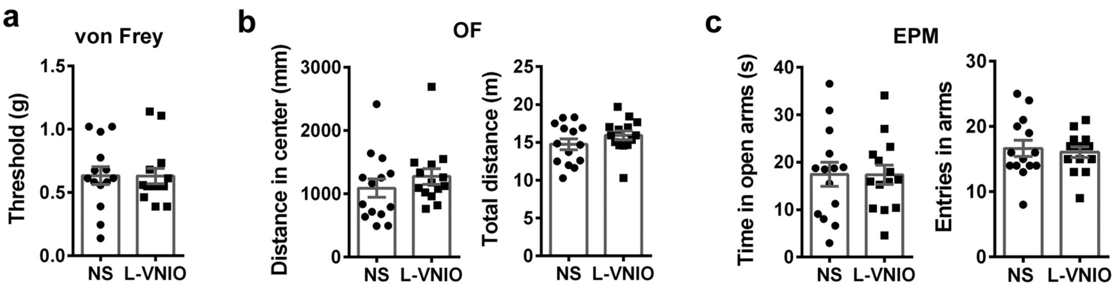

Supplementary Fig. 12 Inhibiting vmPFC nNOS did not produce anxiolytic effects in normal mice. Bar graphs showing withdrawal threshold of hindpaw in von Frey test (**a**), distance in center (**b**, left) and total distiance (**b**, right) in OF, time in open arms (**c**, left) and total entries in arms (**c**, right) of EPM 30 min after L-VNIO (1.5 mM, 1 μl) or NS (1 μl) microinjection into vmPFC. n = 14. Data are mean ± SEM. Source data are provided as a Source Data file. Exact p values and additional statistical information can be found in Source data.

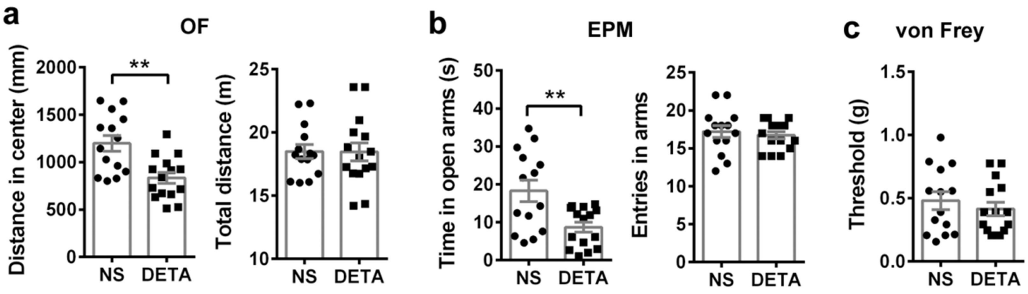

Supplementary Fig. 13 The effect of NO donor microinjection in vmPFC on anxiety-like behaviors. Distance in center (**a**, left) and total distiance (**a**, right) in OF, time in open arms (**b**,

left) and total entries in arms (**b**, right) of EPM, and withdrawal threshold of hindpaw in von Frey test (**c**) 30 min after DETA/NONOate (100 μM, 1 μl) or NS (1 μl) microinjection in vmPFC. *n* = 14 for NS and 15 for DETA. Data are mean ± SEM; ***p* < 0.05 (unpaired two-tailed Student's *t*-test). Source data are provided as a Source Data file. Exact *p* values and additional statistical information can be found in Source data.

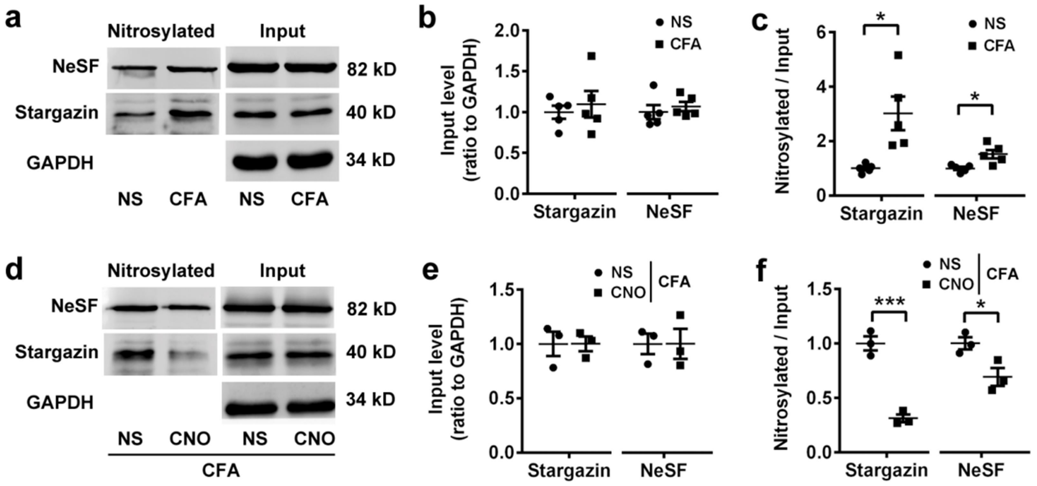

Supplementary Fig. 14 S-nitrosylation of stargazin and NeSF in vmPFC of mice with chronic pain. **a** Representative blots of biotin-switch assay showing vmPFC S-nitrosylated levels of stargazin and NeSF at day 3 after CFA (10 μl) or NS (10 μl) injection. Full-lengthen blots are available in source data online. **b-c** Dot plots for **a**, showing input (**b**) and S-nitrolylated (**c**) levels of stargazin and NeSF. *n* = 5. **d** Representative blots of biotin-switch assay showing S-nitrosylated levels of stargazin and NeSF in vmPFC of nNOS-Cre mice 30 min after CNO (2 mg/kg, ip.) or NS treatment at day 3 after CFA (10 μl) injection (day 21 after 0.4 μl AAV-hSyn-DIO-hM4Di-eGFP microinjection in vmPFC). Full-lengthen blots are available in source data online. **e-f** Dot plots for **d**, showing input (**e**) and S-nitrolylated (**f**) levels of stargazin and NeSF. *n* = 3. Data are mean ± SEM; **p* < 0.05 and ****p* < 0.001 (unpaired two-tailed Student's *t*-test). Source data are provided as a Source Data file. Exact *p* values and additional statistical information can be found in Source data.

Supplementary Discussion

Previously, we demonstrated that hippocampal nNOS-NO is critical for mediating anxiety-related behaviors in mice [1], and we reported an nNOS-related target for developing new anxiolytics [2]. Now, we found that vmPFC nNOS-expressing neurons mediated chronic pain-induced anxiety. This effect is dependent on NO production, but it is independent of classic neurotransmitter (GABA or glutamate) release. Therefore, whether nNOS-expressing neurons in the vmPFC are GABAergic or glutamatergic is not important in the modulation of

chronic pain-induced anxiety. On the other hand, enhanced AMPAR trafficking and functions through S-nitrosylation mediate the downstream molecular mechanism in our study, which is consistent with previous reports about AMPAR changes in the ACC during chronic pain [3] and in the mPFC during chronic pain-induced anxiety [4]. This study provides insight into the cellular and molecular mechanisms of anxiety induced by chronic pain. vmPFC nNOS-expressing neurons are the key for the transformation of signals from chronic pain to anxiety, and NO-dependent AMPAR trafficking may contribute to this transformation.

Traditionally, the PVT is divided into anterior (aPVT, starting at bregma -0.2 mm in mice) and posterior (pPVT, starting at bregma -1.82 mm in mice) subregions [5]. According to three prominent features observed in the obtained images of PVT (the quite large volume of lateral habenula, the presence of the fasciculus retroflexus and the semi-concave shape at the bottom of the PVT), we have targeted pPVT. It was recently reported that two genetically, anatomically and functionally distinct cell types segregate across the anteroposterior axis of the paraventricular thalamus based on their control of arousal and stress [6]. Meanwhile, a series of works from Prof. Chen's lab linking the PVT to chronic pain has mainly implicated the aPVT [7, 8]. However, in our study, we demonstrate a critical role for the pPVT in chronic pain and induced anxiety. How can the discrepancy about subregions be explained? There is a possibility that the whole PVT is implicated in chronic pain; Prof. Chen and his colleagues did not exclude the role of pPVT, and we did not exclude the role of aPVT. In addition, the PVT subregion control of pain may segregate across the anteroposterior axis, making it similar to their control of arousal and stress. Type I neurons (largely restricted to the pPVT) innervate the PL of the mPFC, whereas type II neurons (predominate in the aPVT) mainly target the IL of the mPFC [6]. Whether these anatomical differences would result in distinct control of chronic pain remains unknown.

Supplementary References

1. Zhang J, Huang XY, Ye ML, Luo CX, Wu HY, Hu Y, *et al*. Neuronal nitric oxide synthase alteration accounts for the role of 5-HT1A receptors in modulating anxiety-related behaviors. *J Neurosci* **30**: 2433-2441 (2010).
2. Zhu LJ, Li TY, Luo CX, Jiang N, Chang L, Lin YH, *et al*. CAPON-nNOS coupling can serve as a

target for developing new anxiolytics. *Nat Med* **20**: 1050-1054 (2014).
3. Xu H, Wu LJ, Wang H, Zhang X, Vadakkan KI, *et al*. Presynaptic and postsynaptic amplifications of neuropathic pain in the anterior cingulate cortex. *J Neurosci* **28**: 7445-7453 (2008).
4. Wang GQ, Cen C, Li C, Cao S, Wang N, Zhou Z, *et al*. Deactivation of excitatory neurons in the prelimbic cortex via Cdk5 promotes pain sensation and anxiety. *Nat Commun* **6**: 7660 (2015). doi: 10.1038/ncomms8660. https://www.ncbi.nlm.nih.gov/pmc/articles/PMC4518290/
5. Paxinos G & Franklin KBJ. The Mouse Brain in Stereotaxic Coordinates. 2nd edition, Elsevier Academic Press (2004).
6. Gao C, Leng Y, Ma J, Rooke V, Rodriguez-Gonzalez S, Ramakrishnan C, *et al*. Two genetically, anatomically and functionally distinct cell types segregate across anteroposterior axis of paraventricular thalamus. *Nat Neurosci* **23**: 217-228 (2020).
7. Chang YT, Chen WH, Shih HC, Min MY, Shyu BC, Chen CC. Anterior nucleus of paraventricular thalamus mediates chronic mechanical hyperalgesia. *Pain* **160**: 1208-1223 (2019).
8. Chen WK, Liu IY, Chang YT, Chen YC, Chen CC, Yen CT, *et al*. Ca(v)3.2 T-type Ca2+ channel-dependent activation of ERK in paraventricular thalamus modulates acid-induced chronic muscle pain. *J Neurosci* **30**: 10360-10368 (2010).
