# Supplementary material for: nNOS-expressing neurons in the vmPFC transform pPVT-derived chronic pain signals into anxiety behaviors
Source: Nat Commun. 2020 May 19;11:2501. doi: 10.1038/s41467-020-16198-5 (PMC7237711; doi:10.1038/s41467-020-16198-5)
Supplement: Supplementary file 3 — Reporting Summary [file 41467_2020_16198_MOESM3_ESM.pdf]

nature research

Corresponding author(s): Chun-Xia Luo

Last updated by author(s): Apr 3, 2020

Reporting Summary

Nature Research wishes to improve the reproducibility of the work that we publish. This form provides structure for consistency and transparency in reporting. For further information on Nature Research policies, see Authors & Referees and the Editorial Policy Checklist .

Statistics

For all statistical analyses, confirm that the following items are present in the figure legend, table legend, main text, or Methods section.

n/a	Confirmed	
☐	☒	The exact sample size (*n*) for each experimental group/condition, given as a discrete number and unit of measurement
☒	☐	A statement on whether measurements were taken from distinct samples or whether the same sample was measured repeatedly
☐	☒	The statistical test(s) used AND whether they are one- or two-sided<br>*Only common tests should be described solely by name; describe more complex techniques in the Methods section.*
☒	☐	A description of all covariates tested
☐	☒	A description of any assumptions or corrections, such as tests of normality and adjustment for multiple comparisons
☐	☒	A full description of the statistical parameters including central tendency (e.g. means) or other basic estimates (e.g. regression coefficient) AND variation (e.g. standard deviation) or associated estimates of uncertainty (e.g. confidence intervals)
☐	☒	For null hypothesis testing, the test statistic (e.g. *F*, *t*, *r*) with confidence intervals, effect sizes, degrees of freedom and *P* value noted<br>*Give P values as exact values whenever suitable.*
☒	☐	For Bayesian analysis, information on the choice of priors and Markov chain Monte Carlo settings
☒	☐	For hierarchical and complex designs, identification of the appropriate level for tests and full reporting of outcomes
☒	☐	Estimates of effect sizes (e.g. Cohen's *d*, Pearson's *r*), indicating how they were calculated

Our web collection on statistics for biologists contains articles on many of the points above.

Software and code

Policy information about availability of computer code

Data collection	pClamp 10, Molecular Devices, http://mdc.custhelp.com/, RRID: SCR_011323<br>TopScan LITE software, Clever Sys, Inc., http://www.cleversysinc.com/, RRID: SCR_014494<br>ZEN2009 Light Edition, Carl Zeiss, https://www.zeiss.com/, RRID: SCR_013672<br>Image Lab (Beta 7), Bio-Rad Laboratories, http://www.bio-rad.com/en-us/sku/1709690-image-lab-software/, RRID: SCR_014210
Data analysis	GraphPad Prism 6 software, GraphPad Software, Inc., https://www.graphpad.com/, RRID: SCR_002798<br>pClamp 10, Molecular Devices, http://mdc.custhelp.com/, RRID: SCR_011323<br>Mini Analysis Program 6.0, Synaptosoft, Inc., http://www.synaptosoft.com/MiniAnalysis/, RRID: SCR_002184<br>TopScan LITE software, Clever Sys, Inc., http://www.cleversysinc.com/, RRID: SCR_014494<br>ZEN2009 Light Edition, Carl Zeiss, https://www.zeiss.com/, RRID: SCR_013672<br>Adobe Photoshop CS6, Adobe Systems, https://www.adobe.com/, RRID: SCR_014199<br>Image Lab (Beta 7), Bio-Rad Laboratories, http://www.bio-rad.com/en-us/sku/1709690-image-lab-software/, RRID: SCR_014210<br>ImageTool 3.0, UTHSCSA, http://uthscsa-imagetool.software.informer.com/, RRID:SCR_016208

For manuscripts utilizing custom algorithms or software that are central to the research but not yet described in published literature, software must be made available to editors/reviewers. We strongly encourage code deposition in a community repository (e.g. GitHub). See the Nature Research guidelines for submitting code & software for further information.

Data

Policy information about availability of data

All manuscripts must include a data availability statement. This statement should provide the following information, where applicable:

- Accession codes, unique identifiers, or web links for publicly available datasets
- A list of figures that have associated raw data
- A description of any restrictions on data availability

All the data supporting the findings of this study are available within the paper and its supplementary information file and from the corresponding author upon reasonable request. Source data for all figures are provided with the paper online.

Field-specific reporting

Please select the one below that is the best fit for your research. If you are not sure, read the appropriate sections before making your selection.

[x] Life sciences   [ ] Behavioural & social sciences   [ ] Ecological, evolutionary & environmental sciences

For a reference copy of the document with all sections, see nature.com/documents/nr-reporting-summary-flat.pdf

Life sciences study design

All studies must disclose on these points even when the disclosure is negative.

Sample size	No sample-size calculation was performed. Sample size was 12-16 mice for animal behavioral tests, 3-6 mice for biological measurements and 10-33 neurons from 4-6 mice for electrophysiological recordings. According to previously reported studies in which these experiments were conducted, such sample sizes were sufficient (Science 363: 276-281 (2019); Nat Commun 6: 7660 (2015). doi: 10.1038/ncomms8660; Neuron 87: 605-620 (2015)). Our prior experience and preliminary experimental data also ensured the sufficiency. We have done many works on depression and anxiety behaviors in rodents and data were published (Proc Natl Acad Sci USA 109: 14224-14229 (2010); Nat Med 20: 1050-1054 (2014); J Neurosci 30: 2433-2441 (2010)). In this study, we have obtained positive results in most of our experiments, suggesting the sample size was sufficient to show the difference between groups. Regarding the few negative results, similar sample sizes were used with the experiments in which positive results were obtained. More importantly, our claims were always demonstrated with multiple experiments. The results from different experiments were consistent with each other, indicating that the sample size was sufficient to give true results. False positive or negative results at random could hardly always agree with each other.
Data exclusions	For biological measurements and electrophysiological recordings, no data were excluded from the analysis unless the data were not successfully obtained for technical or accidental reasons. For behavioral tests, animals were excluded from the analysis if accidental events occured, such as escape, fall from the open arms of elevated plus maze, or restless movement in hyperalgesia measurement. The data from the mice whose brain region was not been correctly targeted in microinjection were also excluded. Exclusion criteria were pre-established.
Replication	The accurate target of desired brain regions has been replicated with 10 mice in 10 independent experiments. Representative micrographs shown without statistical quantification were always conducted in triplicate with 3-6 mice and similar results were observed. For animal behavioral tests and other quantified experiments, none was replicated with the same condition except some preliminary experiments for animal behavioral tests. All the findings in the preliminary experiments were successfully replicated by the formal experiments. In the formal experiments, animals or samples were always divided into 2-3 cohorts (each cohort included equal number of animals or samples from all groups) for big sample sizes and received treatments or measurements on different days. Therefore, replication was successfully achieved in once experiment. Additionally, in this study, the most of experimental findings were verified with different methods. For example, the anxiety-like behaviors in OF and EPM were confirmed with NSF and LDB.
Randomization	Random number table was used to randomly allocate animals to different groups.
Blinding	The investigators were blinded to group allocation during data collection and analysis.

Reporting for specific materials, systems and methods

We require information from authors about some types of materials, experimental systems and methods used in many studies. Here, indicate whether each material, system or method listed is relevant to your study. If you are not sure if a list item applies to your research, read the appropriate section before selecting a response.

Materials & experimental systems

n/a	Involved in the study
☐	☒ Antibodies
☒	☐ Eukaryotic cell lines
☒	☐ Palaeontology
☐	☒ Animals and other organisms
☒	☐ Human research participants
☒	☐ Clinical data

Methods

n/a	Involved in the study
☒	☐ ChIP-seq
☒	☐ Flow cytometry
☒	☐ MRI-based neuroimaging

Antibodies

Antibodies used

Rabbit anti-nNOS, polyclonal, Thermo Scientific, Cat# 61-7000, RRID: AB_2313734
Guinea pig anti-c-Fos, polyclonal, Synaptic System, Cat# 226-004, RRID: AB_2619946
Chicken anti-GFP, polyclonal, Millipore, Cat# AB16901, RRID: AB_90890
Rabbit anti-GFP, polyclonal, Abcam, Cat# ab290, RRID: AB_303395
Mouse anti-mCherry, monoclonal, Abcam, Cat# ab125096, Clone 1C51, RRID: AB_11133266
Rabbit anti-CaMKII alpha, polyclonal, Abcam Cat# ab103840, RRID: AB_10900968
Mouse anti-vGAT, monoclonal, Synaptic System, Cat# 131011, Clone 117G4, RRID: AB_887872
Rabbit anti-GluA1, monoclonal, Abcam, Cat# ab109450, Clone EPR5479, RRID: AB_10860361
Rabbit anti-GluA2, monoclonal, Abcam, Cat# ab133477, Clone EPR5032, RRID: AB_2620181
Rabbit anti-stargazin, polyclonal, Millipore, Cat# 07-577, RRID: AB_310726
Rabbit anti-NeSF, polyclonal, Abcam, Cat# ab87155, RRID: AB_10674097
Mouse anti-GAPDH, monoclonal, KangChen Bio-tech, Cat# KC-5G4, Clone 6C5, RRID: AB_2493106
Cy3 goat anti-rabbit, Jackson ImmunoResearch Laboratories, Cat# 111-165-003, RRID: AB_2338000
Alexa 488 goat anti-guinea pig, Abcam, Cat# ab150185, RRID: AB_2736871
Alex 488 goat anti-chicken, Jackson ImmunoResearch Laboratories, Cat# 103-545-155, RRID: AB_2337390
Cy3 goat anti-mouse, Jackson ImmunoResearch Laboratories, Cat# 115-165-003, RRID: AB_2338680
Alexa 488 Goat anti-rabbit, Jackson ImmunoResearch Laboratories, Cat# 111-545-003, RRID: AB_2338046
Goat Anti-mouse HRP, Multi Science, Cat# 70-GAM0072, RRID:AB_2827834

Validation

Validation acronyms: Immunoprecipitation (IP), immunofluorescence (IF), Western-blot (WB), ImmunoHistoChemistry (IHC), Immunoytochemistry (ICC).
-Rabbit anti-nNOS, statement from Thermo Scientific, validated for ELISA, IHC,WB, IF, IM, ICC, IP. (https://www.thermofisher.com/cn/zh/antibody/product/nNOS-Antibody-Polyclonal/61-7000)
-Guinea pig anti-c-Fos, statement from Synaptic System, validated for WB, IP, ICC, IHC, IHC-P/FFPE, FACS. (https://www.sysy.com/products/c-fos/facts-226004.php)
-Chicken anti-GFP, statement from Millipore, validated for ICC, IP, WB. (https://www.merckmillipore.com/CN/zh/product/Anti-Green-Fluorescent-Protein-Antibody,MM_NF-AB16901)
-Rabbit anti-GFP, statement from Abcam, validated for Flow Cyt, ELISA, ICC/IF, ChIP, IHC-FrFl, ChIP/Chip, IHC - Wholemount, Electron Microscopy, IHC-FoFr, ICC, IHC-P, IHC-Fr, IP, WB.
(https://www.abcam.cn/gfp-antibody-chip-grade-ab290.html)
-Mouse anti-mCherry, statement from Abcam, validated for WB, ICC/IF, IHC-Fr, IHC-P.
(https://www.abcam.cn/mcherry-antibody-1c51-ab125096.html)
-Rabbit anti-CaMKII alpha, statement from Abcam,validated for WB, IHC-P.
(https://www.abcam.cn/camkii-alpha-antibody-ab103840.html)
-Mouse anti-vGAT, statement from Synaptic System, validated for WB, IP, ICC, IHC, IHC-P/FFPE, FACS, EM.
(https://www.sysy.com/products/vgat/facts-131011.php)
-Rabbit anti-GluA1, statement from Abcam, validated for IHC-Fr, WB, IP.
(https://www.abcam.cn/glutamate-receptor-1-ampa-subtype-antibody-epr5479-ab109450.html)
-Rabbit anti-GluA2, statement from Abcam, validated for IP, Flow Cyt, WB, ICC/IF.
(https://www.abcam.cn/ionotropic-glutamate-receptor-2-antibody-epr5032-ab133477.html)
-Rabbit anti-stargazin, statement from Millipore, validated for ICC, IP, WB.
(https://www.merckmillipore.com/CN/zh/product/Anti-Stargazin-Cacng2-Antibody-CT,MM_NF-07-577)
-Rabbit anti-NeSF, statement from Abcam, validated for ICC/IF, WB.
(https://www.abcam.cn/n-ethylmaleimide-sensitive-fusion-protein-antibody-ab87155.html)
-Mouse anti-GAPDH, statement from KangChen Bio-tech, validated for ICC/IF, WB, ELISA.
(http://www.bioon.com.cn/antibody/show_product.asp?id=173205)

Animals and other organisms

Policy information about studies involving animals; ARRIVE guidelines recommended for reporting animal research

Laboratory animals	Male young adult (6-7 weeks) C57BL/6 mice (Model Animal Research Center of Nanjing University, Nanjing, China) and nNOS-Cre mice (B6.129-Nos1tm1(cre)Mgmj/J; The Jackson Laboratory; stock number: 017526) were used in this study.
Wild animals	The study did not involved wild animals.
Field-collected samples	The study did not involved the samples collected from the field.
Ethics oversight	All animal experiments were conducted in accordance with the Institutional Animal Care and Use Committee of Nanjing Medical University.

Note that full information on the approval of the study protocol must also be provided in the manuscript.
